# Artesunate Plus Amodiaquine (AS+AQ) Versus Artemether -Lumefantrine (AL) for the Treatment of Uncomplicated Plasmodium Falciparum Malaria in Sub-Saharan Africa-A Meta-Analysis

**DOI:** 10.4314/ajid.v4i2.55149

**Published:** 2010

**Authors:** Shaibu O Bello, Aminu Chika, Jimoh O AbdulGafar

**Affiliations:** 1 Department of Pharmacology, College of Health sciences, Usmanu Danfodiyo University, Sokoto, Nigeria; 2 Fertility Unit, Karaye Hospital, Emir Yahaya Road, Sokoto, Nigeria

**Keywords:** Artemisinin combination therapy, Malaria, Artemether, Artesunate, Amodiaquine, lumefanthrine, plasmodium, meta-analysis

## Abstract

The purpose of this study is to summarize the available data on the efficacy of Artesunate plus Amodiaquine (AS+AQ) versus Artemether -Lumefantrine (AL) for the treatment of uncomplicated *Plasmodium falciparum* malaria in sub-Saharan Africa using uncorrected parasitaemia as a clinically relevant endpoint. Studies and conference abstracts identified through Pubmed, Medline, Embase, Ansinet, AJOL, Bioline, Cochrane Infectious Diseases Group trials register, The Cochrane Controlled Trials Register, Science Citation Index, Lilacs, African Index Medicus, Clusty, Google, Yahoo and Microsoft search engines. Randomized controlled clinical trials comparing Artesunate-Amodiaquine versus Artemether-Lumefantrine, in Sub-Saharan Africa from January 2004 to June 2009, and which had at least 30 patients per study arm. The authors independently applied the inclusion criteria, assessed methodological quality and extracted data into a predesigned form. The outcome of interest was uncorrected day 28 parasitological failure. Data were then checked for agreement and double entered into RevMan version 5 for further analyses. Fifteen trials (4265 participants) met the inclusion criteria. Day 28 parasitological failure was lower for AL (286 of 2201 participants or 13.0 % failures) when compared with AS+AQ (446 of 2424 participants or 18.4% failures). The relative risk of parasitological failure with AS+AQ was higher when compared with AL (RR 1.65, 95% CI, 1.18–2.32). There were significant heterogeneity and inconsistencies in the studies. AL appears more effective at avoiding parasitological failure at days 28 than AS+AQ.

## Introduction

*Plasmodium falciparum* malaria is indigenous between 64°N latitude and 32°S latitude but the main health burden of this disease is borne by sub-Saharan Africa where over 90% of the population is at risk of infection (Snow and Gilles, 2002). In this region of the world, one in every ten deaths in pregnant women and one in every four deaths in under-five children is caused by malaria (Peter et al., 2004). Effective treatment could reduce the morbidity and mortality associated with plasmodium infestation, but high levels of resistance to common antimalarials frequently compromise treatment efforts (Peter et al., 2004; Bello et al., 2005). Choosing a drug with the lowest chance of treatment failure constitutes a particular challenge to clinicians and policy makers (Peter et al., 2004). Potent and rapidly schizonticidal Artemisinin and its derivatives are among the new hope for malarial control. Artemisinin combination therapy (ACT) is the first line treatment recommended by the World Health Organization(WHO) for malaria in endemic regions (WHO, 1998; WHO, 2006). Over 43 countries have accepted this recommendation and 27 countries have now implemented it (WHO, 2007). In these countries, Artesunate-Amodiaquine (AS+AQ) and Artemether-Lumefantrine (AL) are the most common ACTs prescribed for the treatment of malaria and are prescribed as equivalent or non-inferior alternatives (WHO, 2007). Anecdotal evidence suggests that significant and clinically important difference in efficacy exists between them. Most efficacy studies comparing ACT's use polymerase chain reaction(PCR) corrected day 28 and day 43 parasitological clearance as important end points because these days are considered to give good estimates of disease free periods after treatment. Also, PCR correction is considered to give a true estimate of the efficacy of treatment because it sufficiently separates re-infection from recrudescence (Whitty and Staedke, 2005). It may be argued that PCR corrected results are not helpful as aids to decisions at points of treatment because uncorrected parasitaemia (in the form of simple microscopy of blood smears) is the clinical tool used to take decisions as to the need to declare cure, clinical resistance and/or switch therapy (Whitty and Steadke, 2005). Also, parasitaemia, irrespective of whether it is due to re-infection or recrudescence, constitutes ongoing risk of clinical disease. Uncorrected parasitaemia actually estimates the efficacy of treatment against the pre-treatment parasitaemia and post treatment prophylaxis (i.e. ability to prevent re-infection and recrudescence). It has been shown that parasitological failure after ACT tends to occur after day 21 (Ashley and White, 2005). It may, therefore, be important to compare ACT's using uncorrected parasitaemia at days 28 as an index of the probability of declaring clinical cure or otherwise . An ACT that performs better in this regard may claim clinically important superiority. This meta-analysis was carried out to compare AS+AQ and AL using uncorrected day 28 parasitaemia in order to serve as additional guide to contemporary treatment of *Plasmodium falciparum* malaria in Sub-Saharan Africa.

## Methodology

Given that drug efficacy changes over time due to resistance, only trials within January 2004 to June 2009 were considered. Each reviewer independently searched Pubmed, Medline, Embase, Ansinet, African Journal Online (AJOL), Bioline, Cochrane Infectious Diseases Group trials register, the Cochrane Controlled Trials Register, Science Citation Index, Lilacs, African Index Medicus and conference abstracts for randomized controlled clinical trials comparing Artesunate-Amodiaquine versus Artemether-Lumefantrine , in Sub-Saharan Africa from January 2004 to June 2009. The search terms included Artesunate, Artemether, Lumefantrine, Amodiaquine, Artemisinin Combination therapy, ACT, AS+AQ and AL . No language restriction was used. The search was further limited to Sub-Saharan Africa, Clinical trials, letters and randomized controlled trials. To capture as many trials as possible, free search was also performed on Clusty, Google, Yahoo and Microsoft search engines. We also search country specific medical websites using the World Bank database listing the countries in Sub-Saharan Africa as a guide (Worldbank, 2007). The bibliographies of relevant papers were also searched. Information about unregistered and unpublished trials, were obtained by contacting the pharmaceutical industry and selected authors. Grading of allocation concealment was based on the Cochrane approach (adequate, uncertain, and clearly inadequate) (Cochrane Collaboration, 2003). The Jadad scale was used to score overall study quality (Jadad et al., 1996). Studies were considered eligible for inclusion if they were RCTs that included data comparing Artesunate-Amodiaquine versus Artemether-Lumefantrine, the study subject and location was in sub-Saharan Africa, had at least 30 patients per group, was concluded within January 2004 and June 2009 and scored at least 3 on the Jadad scale. The abstract of all trials identified to meet the inclusion criteria were independently examined and the outcomes of interest were recorded into predesigned electronic and paper data abstraction forms. Data were then checked for agreement and entered into RevMan version 5 (Cochrane Collaboration, 2003) for further analyses. Day 28 parasitological failure was the only outcome for meta-analysis.

We used a random effect model to report the difference in outcome between the treatment groups as relative risks (RR) with 95% confidence intervals. We assessed heterogeneity and the extent of inconsistency using chi-square (X^2^) and Inconsistency ( I^2^ ) respectively. We hypothesized that the pre treatment probability of uncorrected day 28 parasitological failure will be expected to depend on the rate of re-infection which is expected to be different between studies and between study sites. This may introduce non random differences in treatment outcome which will cause significant but understandable heterogeneity. We also predicted that spurious heterogeneity may be introduced by studies with outlier outcome especially if heavily weighted. We accept that a fixed effect model may be used in this case because it makes heterogeneity irrelevant, however, it may also make any conclusion difficult to generalize and therefore not plausible to support clinical decisions. We, therefore, maintained a random effect model but pre-specified that, should significant heterogeneity arise, it will be explored by excluding studies with outlier outcome and which also weighed 15% or more in the primary analyses in reducing order (removing the most heavily weighed first). We also pre-specified to explore heterogeneity by running region and country specific as well as timed (3-years interval) analyses and then report all head to head. Should no significant heterogeneity exist in the primary analyses, we pre-specified to do sensitivity analyses using all the criteria previously listed and using subjects aged below or above 5 years. We considered a P value less than 0.05 as significant.

## Results

**Figure 1:**
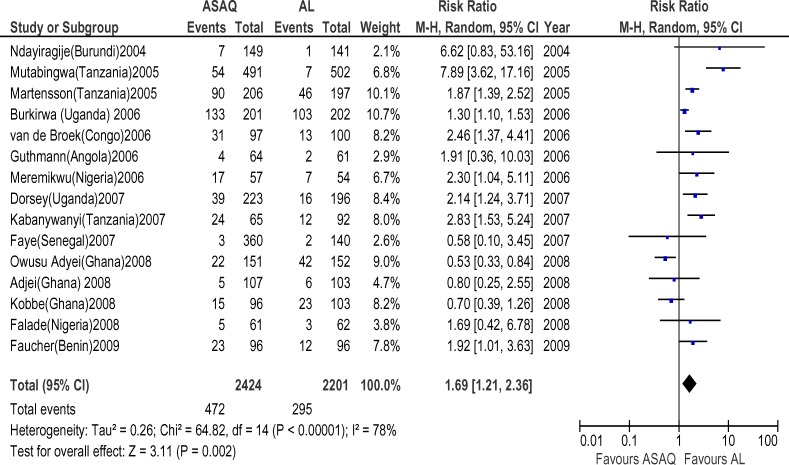
Meta-analysis of Artesunate + Amodiaquine (AS+AQ) Versus Arthemeter-Lumefantrine (AL) All studies that satisfied the inclusion criteria were included in this analysis. The outcome measure of interest (day 28 parasitological failure) is not clinically desirable; therefore, the right side of the graph favours Arthemeter-Lumefantrine. The high level of heterogeneity and Inconsistencies (78%) would seem to require exploration.

**Figure 2:**
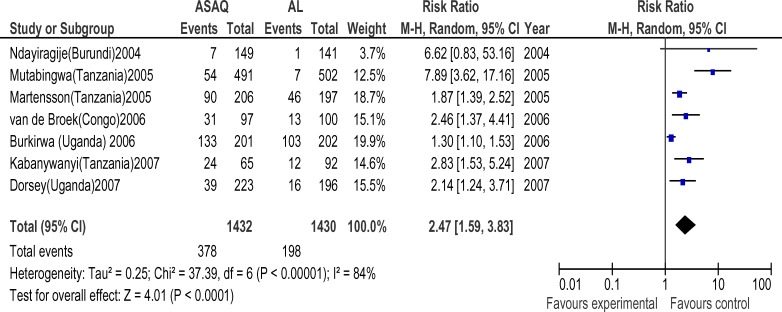
Meta-analysis of Artesunate + Amodiaquine (AS+AQ) Versus Arthemeter-Lumefantrine (AL) in East Africa All studies that satisfied the inclusion criteria and were conducted in East Africa were included in this analysis. The outcome measure of interest (day 28 parasitological failure) is not clinically desirable; therefore, the right side of the graph favours Arthemeter-Lumefantrine.

**Figure 3:**
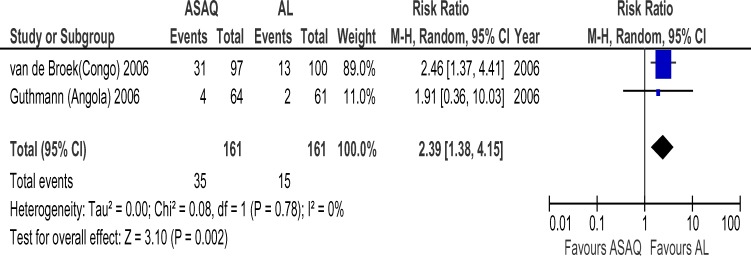
Meta-analysis of Artesunate + Amodiaquine (AS+AQ) Versus Arthemeter-Lumefantrine (AL) in Middle Africa All studies that satisfied the inclusion criteria and were conducted in Middle Africa were included in this analysis. The outcome measure of interest (day 28 parasitological failure) is not clinically desirable; therefore, the right side of the graph favours Arthemeter-Lumefantrine. The absence of inconsistencies is striking

**Figure 4:**
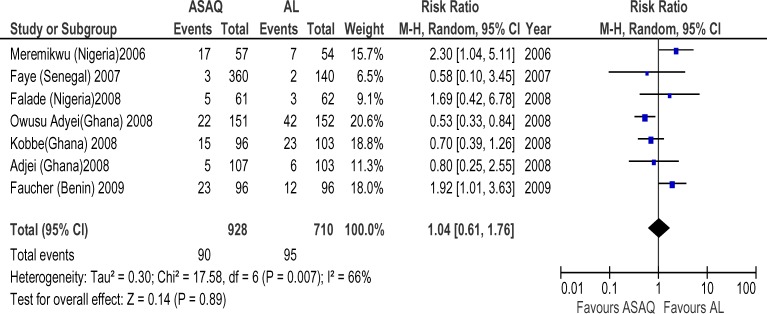
Meta-analysis of Artesunate + Amodiaquine (AS+AQ) Versus Arthemeter-Lumefantrine (AL) in West Africa All studies that satisfied the inclusion criteria and were conducted in West Africa were included in this analysis. The outcome measure of interest (day 28 parasitological failure) is not clinically desirable; therefore, the right side of the graph favours Arthemeter-Lumefantrine.

**Figure 5:**
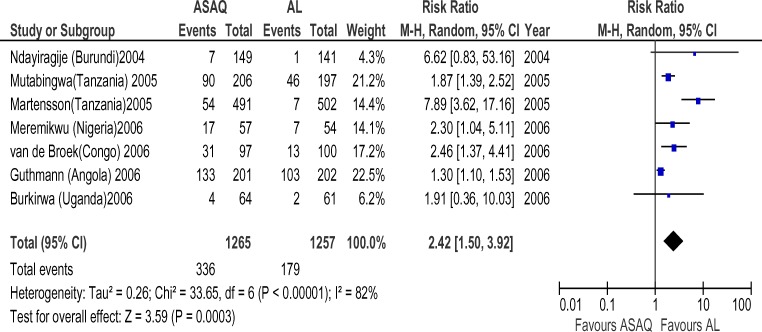
Meta-analysis of Artesunate + Amodiaquine (AS+AQ) Versus Arthemeter-Lumefantrine (AL) (2004–2006) All studies that satisfied the inclusion criteria and were conducted within January 2004 and December 2006 were included in this analysis. The outcome measure of interest (day 28 parasitological failure) is not clinically desirable; therefore, the right side of the graph favours Arthemeter-Lumefantrine.

**Figure 6:**
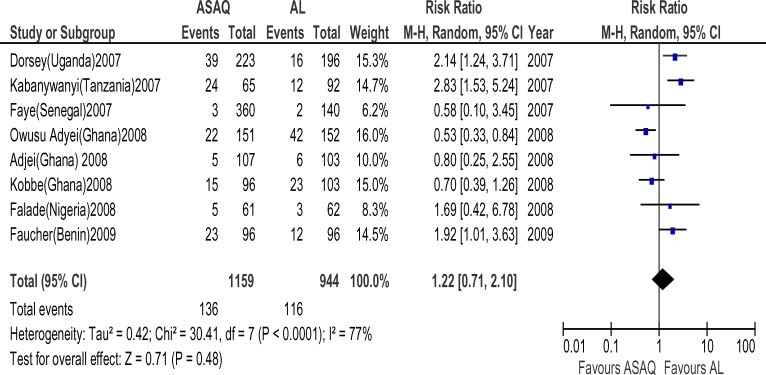
Meta-analysis of Artesunate + Amodiaquine (AS+AQ) Versus Arthemeter-Lumefantrine (AL) (2007–2009) All studies that satisfied the inclusion criteria and were conducted within January 2007 and June 2009 were included in this analysis. The outcome measure of interest (day 28 parasitological failure) is not clinically desirable; therefore, the right side of the graph favours Arthemeter-Lumefantrine.

**Figure 7:**
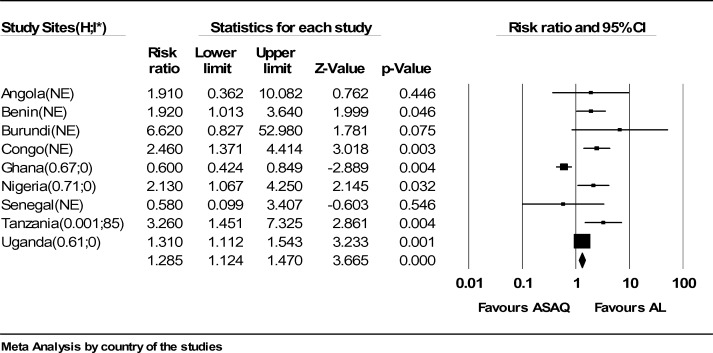
Meta-analysis of Artesunate + Amodiaquine (AS+AQ) Versus Arthemeter-Lumefantrine (AL) according to country. All studies that satisfied the inclusion criteria were included in this analysis. The outcome measure of interest (day 28 parasitological failure) is not clinically desirable; therefore, the right side of the graph favours Arthemeter-Lumefantrine.

**Figure 8:**
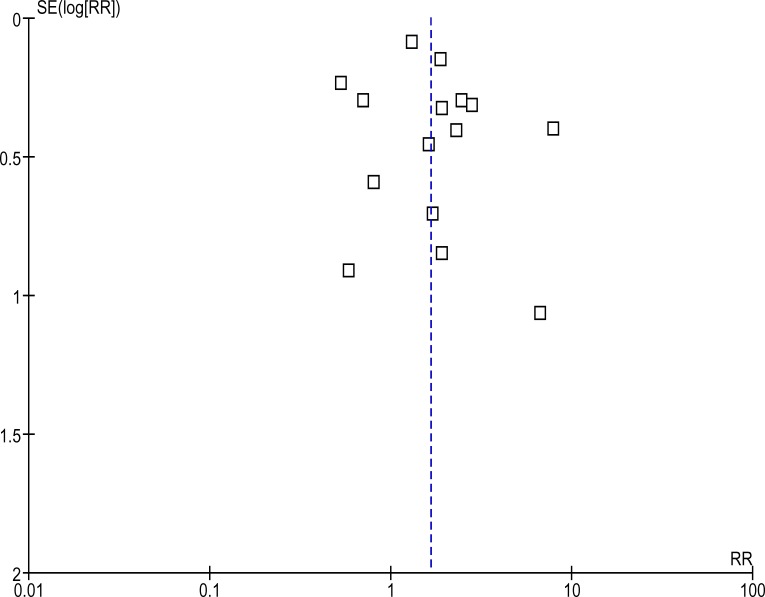
Funnel plot of the pooled studies All studies that satisfied the inclusion criteria were included in this analysis. The outcome measure of interest (day 28 parasitological failure). The symmetry about the no effect (dotted) line appears satisfactory and suggests lack of publication bias.

Forty six randomized trials and 12 conference abstracts that potentially met our inclusion criteria were identified. Twenty trials were excluded because one of the ACT's of interest was not included. Nine trials and all the conference abstracts were excluded because they had less than 19 patients per group or were duplicates of published studies. Two trials were excluded because of the two arms were in different countries. Fifteen trials finally contributed to the analysis, with 4265 subjects. The AS+AQ arm had 2424 patients with 472(19.5%) day 28 parasitological failure while the AL arm had 2201 patients with 295(13.4%) failures and risk ratio of 1.69 ( 95% CI, 1.21–2.36) (Figure 1). There was significant heterogeneity in the study ( X^2^ = 64.82, df= 14 (P=0.00001), I^2^=78%). Analyses according to WHO geographical categorization revealed a risk ratio of day 28 parasitological failure (ASAQ/AL) of 2.47(95% CI , 1.59–3.83) with significant heterogeneity (P=0.00001) and inconsistency(I2=84%) in East Africa (WHO code 002/014), risk ratio of 2.39(95% CI , 1.38–4,15) in Middle Africa (WHO code 002/017) but with no significant heterogeneity (P=0.78) and no inconsistency (I^2^=0%)(Figures 2 and 3 respectively) a . In West Africa (WHO code 002/011) there was no significant difference in risk of day 28 parasitological failure between both ACT (RR:1.04, 95% CI, 0.61–1.71)(Figure 4). Pooled analysis of studies in Tanzania, Nigeria and Uganda revealed risk ratio (ASAQ/AL) of 3.26(95% CI, 1.451–7.235), 2.13(95% CI, 1.067–4.25) and 1.31( 95% CI,1.112–1.543), with no significant heterogeneity and no inconsistency in the last two countries. Conversely, pooled analysis of the Ghanaian studies revealed a significant reduction in risk of day 28 parasitological failure (ASAQ/AL: RR: 0.60, 95% CI, 0.424–0.849) with no significant heterogeneity nor inconsistency . Temporal analysis comparing studies in the first 3 years(2004–2006) (Figure 5) and the last 3 years (2005–2009) (Figure 6) revealed a risk ratio of 2.42(95% CI, 1.50–3.92) with ASAQ/AL in the first 3 years and no significant difference in risk in the last 3 years(RR:1.22, 95% CI, 0.71–2.10). Analyses according to country (Figure 7) revealed only one high quality RCT per country in Angola, Benin, Burundi, Congo and Senegal with all but Senegal showing significantly high risk of parasitological failure of ASAQ over AL .The funnel plot (Figure 8) showed symmetry about the no effect line and showed high precision in even smaller studies. Sensitivity analysis revealed no significant difference in response between subjects aged 5 and below versus subjects aged above 5 years.

## Discussion

This meta-analysis favors AL as the ACT with significantly lower day 28 parasitological failure when compared with AS+AQ, and therefore the combination less likely to require a need to continue treatment. This finding is clinically important in Sub-Saharan Africa where follow up on treatment may be difficult. The significant heterogeneity is a drawback of this conclusion. The heterogeneity is probably a clinical one given that the micro-ecology of malaria is known to vary. The pooled analysis using both the WHO geographical categorization and country of study is quite revealing. Except Ghana and Tanzania, studies in all other countries are consistent in suggesting that AL has lower risk of day 28 parasitological failure. The result of country specific analyses may be more useful for point of treatment decisions when compared to regional analyses. Other factors that may cause heterogeneity which are not addressed in these analyses include country specific treatment policy and drug quality.

Most studies in the years 2004–2006 were done in East Africa while most studies in 2005–2009 were in West Africa. A publication bias may exist in this regard and may explain the change in risk ratios when these times were compared. The fact that funnel plots may not reveal such publication bias has been described (Sterne et al., 2000). Another reason for the shift in risk ratio over time is increasing resistance. Anecdotal evidence suggest that AL is the most often prescribed ACT mainly because of an estimated higher risk of neurological side effect with ASAQ. This may explain a shift in failures rates towards AL.

The low number of high quality studies comparing AS+AQ and AL is not reassuring. Except in Ghana, Nigeria, Tanzania and Uganda, only one or no high quality RCT has been published in all other Sub-Saharan countries. This suggests that there is little evidence for therapeutic decisions in these countries other than country policies, which may be unfounded.

## Conclusions

Using uncorrected day 28 parasitological failure as the outcome measure, AL has significantly lower risk of failure and may be preferred when compared with AS+AQ. There appears to be few high quality studies comparing AS+AQ and AL head to head with most studies coming from East and West Africa. Malaria is a preventable cause of morbidity and mortality. More quality studies are needed in order to provide robust evidence for competing clinical decisions.
